# Current advances in remote and unsupervised digital cognitive assessment in preclinical Alzheimer’s disease

**DOI:** 10.1002/alz.089046

**Published:** 2025-01-03

**Authors:** Sarah E. Polk, Fredrik Öhman, Jason J. Hassenstab, Alexandra König, Kathryn V. Papp, Michael Schöll, David Berron

**Affiliations:** ^1^ German Center for Neurodegenerative Diseases (DZNE), Magdeburg Germany; ^2^ Institute of Neuroscience and Physiology, Sahlgrenska Academy, University of Gothenburg, Gothenburg Sweden; ^3^ Wallenberg Centre for Molecular and Translational Medicine, University of Gothenburg, Gothenburg Sweden; ^4^ Washington University in St. Louis, St. Louis, MO USA; ^5^ Washington University in St. Louis, School of Medicine, St. Louis, MO USA; ^6^ ki elements UG, Saarbrücken Germany; ^7^ National Institute for Research in Computer Science and Automation (INRIA), Sophia Antipolis France; ^8^ Brigham and Women’s Hospital, Harvard Medical School, Boston, MA USA; ^9^ Massachusetts General Hospital, Harvard Medical School, Boston, MA USA; ^10^ University of Gothenburg, Gothenburg Sweden; ^11^ Clinical Memory Research Unit, Lund University, Lund Sweden

## Abstract

**Background:**

Traditional pen‐and‐paper neuropsychological assessments fail to capture subtle cognitive changes in the early stages of Alzheimer’s disease (AD). Remote and unsupervised digital assessments available on smartphones, tablets, and personal computers may offer a solution to this by increasing the amount and types of data available to researchers and clinicians, while simultaneously improving ecological validity and alleviating patient burden. As these remote and unsupervised digital cognitive assessment tools become more widely available, it is important that they are validated in a systematic way. In this review, we evaluate the validity of available remote tools, focusing on active assessment tools with which the patient interacts with (i.e., cognitive tests, active speech) that have been used to investigate subtle cognitive differences in preclinical AD, defined as clinically unimpaired individuals with biomarkers indicating increased risk of dementia due to AD.

**Methods:**

We performed a systematic literature review on PubMed, Web of Science, and PsycInfo using terms such as “digital,” “remote,” “unsupervised,” “cognition,” “aging,” and “Alzheimer’s,” which resulted in a total of 1,453 unique peer‐reviewed articles and pre‐prints. After filtering for tools that were remotely self‐administered in humans, and excluding papers detailing findings in populations with other diseases than AD (e.g., multiple sclerosis, Parkinson’s disease) or papers reporting findings of intervention studies, 14 papers reporting the use or planned use of 14 tools to detect AD pathology in preclinical AD were selected (as of Jan. 2024).

**Results:**

We evaluate each tool with regards to its use‐case and usability, as well as various types of validity, and suggest a framework with which future tools may also be evaluated. Additionally, we discuss current directions in the field of remote and unsupervised digital cognitive assessment in early AD.

**Conclusion:**

With this review, we hope to show that the systematic and validated use of such tools will increase our understanding of subtle changes in cognition due to AD pathology, as well as make screening for clinical trials and treatment more accessible.

